# Gender Differences in Clinical Presentation and Outcomes of Epidemic Kaposi Sarcoma in Uganda

**DOI:** 10.1371/journal.pone.0013936

**Published:** 2010-11-12

**Authors:** Warren Phipps, Fred Ssewankambo, Huong Nguyen, Misty Saracino, Anna Wald, Lawrence Corey, Jackson Orem, Andrew Kambugu, Corey Casper

**Affiliations:** 1 Department of Medicine, University of Washington, Seattle, Washington, United States of America; 2 Department of Laboratory Medicine, University of Washington, Seattle, Washington, United States of America; 3 Department of Epidemiology, University of Washington, Seattle, Washington, United States of America; 4 Department of Global Health, University of Washington, Seattle, Washington, United States of America; 5 Vaccine and Infectious Disease Division, Fred Hutchinson Cancer Research Center, Seattle, Washington, United States of America; 6 Infectious Diseases Institute, Kampala, Uganda; 7 Uganda Cancer Institute, Kampala, Uganda; 8 College of Health Sciences, Makerere University, Kampala, Uganda; Tulane University, United States of America

## Abstract

**Introduction:**

The incidence of Kaposi sarcoma (KS) has increased dramatically among women in sub-Saharan Africa since the onset of the HIV pandemic, but data on KS disease in women are limited. To identify gender-related differences in KS presentation and outcomes, we evaluated the clinical manifestations and response in men and women with AIDS-associated KS in Uganda.

**Methods and Findings:**

HIV-infected adults with KS attending the Infectious Diseases Institute (IDI) and Uganda Cancer Institute (UCI) in Kampala, Uganda between 2004 and 2006 were included in a retrospective cohort. Evaluation of KS presentation was based on the clinical features described at the initial KS visit. Response was evaluated as the time to “improvement”, as defined by any decrease in lesion size, lesion number, or edema. The cohort consisted of 197 adults with HIV and KS: 55% (108/197) were women. At presentation, the median CD4 T-cell count was significantly lower in women (58 cells/mm^3^; IQR 11–156 cells/mm^3^) than men (124 cells/mm^3^; IQR 22–254 cells/mm^3^) (p = 0.02). Women were more likely than men to present with lesions of the face (OR 2.8, 95% CI, 1.4, 5.7; p = 0.005) and hard palate (OR 2.0, 95% CI, 1.1, 3.7; p = 0.02), and were less likely than men to have lower extremity lesions (OR 0.54, 95% CI, 0.3, 0.99; p = 0.05). Women were less likely than men to demonstrate clinical improvement (HR = 0.52, CI 0.31, 0.88; p = 0.01) in multivariate analysis.

**Conclusions:**

The clinical presentation and response of KS differs between men and women in Uganda. These data suggest that gender affects the pathophysiology of KS, which may have implications for the prevention, diagnosis, and treatment of KS in both men and women. Prospective studies are needed to identify predictors of response and evaluate efficacy of treatment in women with KS, particularly in Africa where the disease burden is greatest.

## Introduction

Kaposi sarcoma (KS) is the most common HIV-related malignancy worldwide and the most frequently diagnosed cancer in several African countries. Previously recognized as a disease almost exclusively of men, the incidence of KS has increased exponentially in women since the beginning of the HIV pandemic, most dramatically among women in sub-Saharan Africa. Prior to the onset of HIV, women accounted for 5–10% of KS cases but now account for up to 40% of incident KS in many African countries [1]–[5]. In Uganda, which has one of the highest rates of KS in the world, the incidence of KS has become nearly equal in men and women, and it has surpassed cervical cancer as the most common female malignancy in the entire population [6].

Despite the increasing burden of disease, little is known about KS in women. Because KS has historically been a male disease and cases in HIV-infected women in the developed world are rare, studies of KS have been predominantly in men [7]. A few reports suggest that epidemic (or HIV-associated) KS in women is associated with more severe disease and worse prognosis compared to men [8]–[11], but data on gender differences in KS are limited, particularly in regions of the world with high burdens of KS. The two published studies describing KS presentation in African women found that they are younger at time of presentation, have more extensive cutaneous disease, and more systemic symptoms than men [12], [13]. However, neither study evaluated clinical outcomes, which could have important implications for the management of KS in African women. We hypothesized that the clinical presentation and outcomes of KS differ by gender in Uganda; to address our hypothesis, we conducted a retrospective study of men and women with HIV-associated KS.

## Methods

### Study Population

We evaluated a cohort of patients with HIV-associated KS who had received HIV care at the Infectious Diseases Institute (IDI) in Kampala, Uganda between January 1, 2004 and December 31, 2006. Patients were eligible for the study if they had histologically or clinically diagnosed KS, had HIV infection, and were ≥18 years of age at the time of KS diagnosis. Only those patients with at least one follow-up clinic visit after their initial KS diagnosis were included in analysis of clinical response.

### Data Collection

Data were obtained by chart review using a standardized case report form. Data were abstracted from both IDI charts and from records at the Uganda Cancer Institute (UCI) in Kampala, where patients from the IDI are referred for cancer care. We linked patient records from the IDI and UCI to compile demographic data and descriptions of KS clinical presentation and outcomes from both institutions.

### Definitions

KS presentation was based on variables described at the initial KS visit at the IDI and UCI. Demographic variables included patient age at KS diagnosis and gender. Baseline clinical characteristics included body mass index (BMI), Karnofsky score, and CD4 T-cell count. BMI was categorized as underweight if BMI<18.5 kg/m^2^ and normal weight if BMI ≥18.5 kg/m^2^ [14]. The Karnofsky score, a measure of performance status, was categorized as a score ≥70 and <70 to distinguish those who can and cannot care for themselves without assistance [15]. The CD4 T-cell count obtained closest to the KS diagnosis and within 6 months of diagnosis was used in the analyses. CD4 count was evaluated as both a continuous and dichotomous variable, categorized as <200 and ≥200 cell/ml. Tumor stage was defined according to the AIDS Clinical Trials Group (ACTG) classification as T1 if there was visceral involvement, tumor-associated edema or ulcerations, or oral involvement beyond the hard palate; otherwise, stage was classified as T0 [16]. Lesion morphotype was classified as “macular”, “nodular” or “fungating” if explicitly described as such in the medical record. Anatomic sites were categorized as face/neck, hard palate, oral (outside hard palate), trunk, upper extremities, lower extremities, and genitals.

For evaluation of response, “improvement” was defined as any decrease in lesion size, lesion number, or lesion-associated edema noted in the clinical charts, to reflect AIDS Clinical Trials Group (ACTG) KS response criteria [17]. “Time to improvement” was calculated from the date of KS diagnosis to the date of the first clinic visit that the patient met criteria for “improvement”.

### Statistical Analysis

To explore our hypothesis that KS clinical presentation differs between men and women, we evaluated patient characteristics at KS diagnosis, including age, BMI, Karnofsky score, CD4 count, tumor stage, lesion morphotype, lesion location, lesion number, and tumor-associated edema. Differences in KS presentation were determined using Pearson's chi-square test and logistic regression.

To evaluate our hypothesis that clinical outcomes differ by gender, we estimated the cumulative incidence of disease improvement using Kaplan-Meier survival analysis. Events were censored at the date of the last visit, date of death, at 180 days, or at the end of the study period (December 31, 2006), whichever came first. Observations were censored at 180 days (6 months) to focus on gender differences in the period immediately following presentation and to limit the potential effect of patients with longer follow-up on the overall risk estimates. We fitted a Cox proportional hazard model for time to improvement for the same demographic and clinical covariates assessed in KS presentation. Univariate predictors with a two-sided p-value ≤0.2 were included in the multivariate model, and backward elimination was applied to obtain a final model; two-sided p-values ≤0.05 were considered significant. Because this retrospective study includes a fixed sample size, associations not demonstrated should not be viewed as definitive lack of association. To test our hypotheses about gender differences in KS with our cohort sample size, we have 80% power to recognize a 53% difference in the frequency of clinical characteristics at presentation and a 59% difference in the hazard of improvement between men and women with a significance level of 0.05. All statistical calculations were performed using Stata 11.0 (Statacorp, College Station, TX).

### Ethics Statement

The Institutional Review Boards at Makerere University, Mulago Hospital, the Uganda National Council for Science and Technology, and the University of Washington approved the study procedures. The Institutional Review Boards determined that this retrospective study met requirements for a waiver of consent and authorization.

## Results

### Characteristics of Study Cohort

We identified 197 adults who attended the IDI with a diagnosis of HIV-associated KS between January 2004 and December 2006. Women comprised 55% of the study cohort (**Table 1**). The median age was 35 years (range, 21–57 years) among men and 34 years (range, 18–61 years) among women (p = 0.14). Most patients had a Karnofsky score ≥70% at the time of KS diagnosis (78%), and 65 (33%) were considered underweight with a BMI of less than 18.5 kg/m^2^; Karnofsky score and BMI did not differ significantly between men and women. Among the 153 (78%) patients with a CD4 T-cell count documented within 6 months of KS diagnosis, the median CD4 count was 84 cells/mm^3^ (IQR: 14–208 cells/mm^3^). The CD4 count was significantly lower in women (median 58 cells/mm^3^; IQR: 11–156 cells/mm^3^) than men (median 124 cells/mm^3^; IQR: 22–254 cells/mm^3^) (p = 0.02).

**Table 1:** Characteristics of Study Population, by Gender (N = 197)

Characteristic	Total Cohort (N = 197)	Men (N = 89)	Women (N = 108)	P-value^1^
**Age at KS Diagnosis** (median, range)	35 (18, 61)	35 (21–57)	34 (18–61)	0.18
**BMI**:				
Underweight (<18.5 kg/m^2^)	65 (33%)	25 (28%)	40 (37%)	0.30
Normal weight (≥18.5 kg/m^2^)	97 (49%)	45 (51%)	52 (48%)	
Missing	35 (18%)	19 (21%)	16 (15%)	
**Karnofsky Score:**				
<70	30 (15%)	14 (16%)	16 (15%)	0.85
≥70	154 (78%)	69 (77%)	85 (79%)	
Missing	13 (7%)	6 (7%)	7 (6%)	
**Baseline CD4 Count** (n = 153)^2^ (<6 mos of KS dx), Median (IQR)	84 (14, 208)	124 (22,254)	58 (11, 156)	**0.01**
**Tumor Stage**:				
T0	66 (34%)	29 (33%)	37 (34%)	0.50
T1	116 (59%)	57 (64%)	59 (55%)	
Not specified	15 (7%)	3 (3%)	12 (11%)	
**Number of Locations Involved**				
1	81 (41%)	41 (46%)	40 (37%)	0.40
2 or more	97 (49%)	43 (48%)	54 (50%)	
Missing	19 (10%)	5 (6%)	14 (13%)	
**Number of Lesions**				
<10	68 (35%)	32 (36%)	36 (33%)	0.77
≥10	43 (22%)	19 (21%)	24 (22%)	
Missing	86 (44%)	38 (43%)	48 (44%)	
**Lesion-associated Edema**				
Edema	67 (34%)	41 (46%)	26 (24%)	**0.002**
No edema	127 (65%)	48 (54%)	79 (73%)	
Missing	3 (1%)	0 (0%)	3 (3%)	

1 P-values reflect presence of characteristic in men vs. women, calculated using logistic regression. Calculations exclude “Missing” or “Not Specified” data. P-value for lesion type and location reflect presence of listed characteristic in men vs women.

2 N = 153 (78%) with CD4 documented within 6 months of KS diagnosis.

### KS Clinical Presentation

KS diagnosis was confirmed by histology in 123 (62%) of the patients; the rest of the cases were diagnosed clinically. Sixty-six (34%) patients were classified as T0 tumor stage and 116 (59%) were classified as T1 at KS diagnosis. Tumor stage did not differ significantly between men and women.

Lesions involved the following locations among patients at KS presentation: 47 (24%) face or neck; 69 (35%) hard palate; 30 (15%) oral cavity outside hard palate; 43 (22%) trunk; 43 (22%) upper extremities; 125 (63%) lower extremities; 11 (6%) genitals. Ninety-seven (49%) patients had KS lesions involving 2 or more anatomic locations. The median number of lesions per patient was 7 (range, 1–85 lesions).

**Figure 1:**
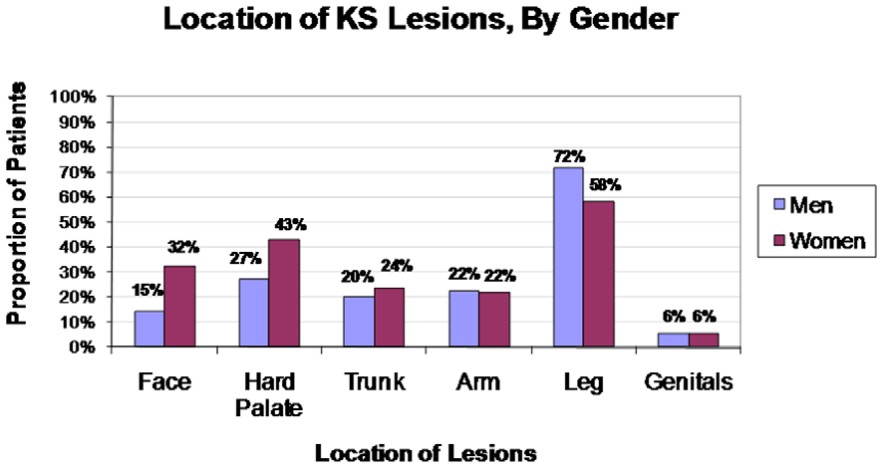
Location of KS Lesions By Gender. Women were more likely than men to have KS lesions involving the face (32% vs 15%, p = 0.005) and the hard palate (43% vs 27%, p = 0.02). Women were less likely than men to have lower extremity lesions (58% vs 72%, p = 0.05).

Women were more likely than men to present with lesions involving the face or neck (OR 2.8, 95% CI, 1.4, 5.7; p = 0.005) and hard palate (OR 2.0, 95% CI, 1.1, 3.7; p = 0.02). Women were less likely than men to have lower extremity lesions (OR 0.54, 95% CI, 0.30, 0.99; p = 0.05) (**Figure 1**). The greater likelihood of lesions involving the face or neck among women remained significant after adjusting for CD4 count. There were no gender differences in the number of involved anatomic locations or the total number of lesions at presentation.

**Figure 2:**
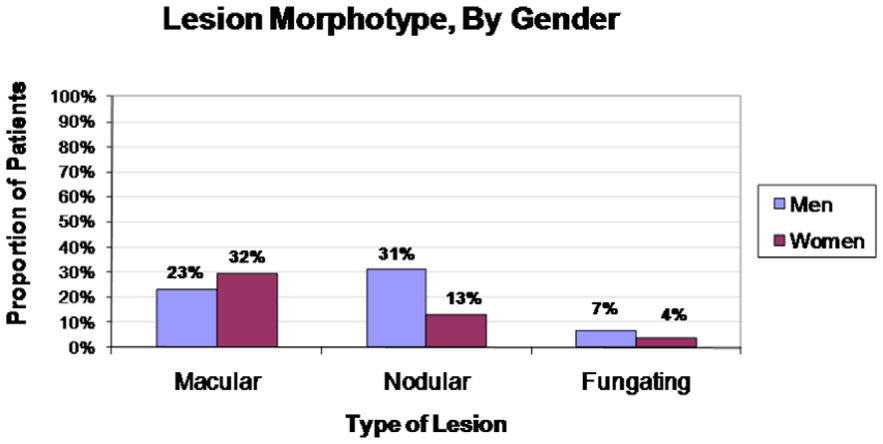
Types of KS Lesions By Gender. Women were significantly less likely to present with nodular KS lesions compared to men (13% vs 31%, p = 0.003), (N = 99).

Fifty-one (26%) patients had macular lesions, 42 (21%) had nodular lesions, 10 (5%) had fungating lesions, and 98 (50%) did not have lesion morphotype documented (**Figure 2**). Only 7 (3%) had more than one type of lesion. Women were less likely than men to have nodular lesions (OR 0.33, 95% CI, 0.16, 0.69; p = 0.003), which remained significant after adjusting for CD4 count and lesion location.

Edema was associated with KS lesions in 67 (34%) patients. Women were less likely than men to have edema at any location (OR 0.38, 95% CI, 0.21, 0.71; p = 0.002). This difference was attenuated slightly after adjusting for CD4 count and lower extremity involvement (OR 0.45, 95% CI, 0.19, 1.05; p = 0.06).

### Clinical Response

177 (90%) of 197 patients had at least 1 follow-up visit after diagnosis of KS, of whom 84 (47%) were men and 93 (53%) were women. During the 6-month period of follow up, 68 (38%) of the 177 patients had improvement in KS disease. The 6-month cumulative incidence of improvement was 0.50 (95% CI 0.41, 0.58).

**Figure 3:**
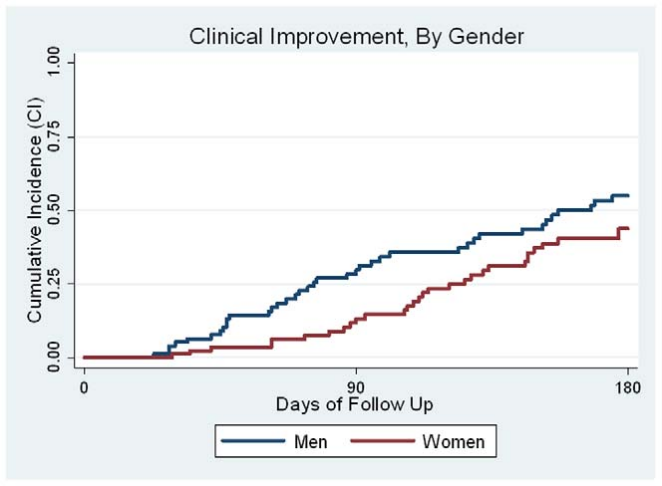
Cumulative Incidence of Improvement By Gender. Univariate analysis (HR = 0.64, CI 0.40, 1.04; p = 0.07).

Women were less likely to demonstrate clinical improvement than men (**Figure 3**). This finding was near statistical significance in univariate analysis (HR = 0.64, CI 0.40, 1.04; p = 0.07) and significant in multivariate analysis, with women having a 0.52 fold decreased probability of clinical improvement compared to men (CI 0.31, 0.88; p = 0.01). Presenting with lesions located on the hard palate was associated with a 2.0 fold greater probability of improvement (CI 1.2, 3.4; p = 0.008) and on the trunk with a 1.7 fold greater probability of improvement (CI 1.0, 2.8; p = 0.04) in the multivariate model.

No other variables, including age, BMI, Karnofsky score, baseline CD4 count, tumor stage, lesion location, number of lesions, or presence of edema were found to be associated with likelihood of improvement in multivariate analysis.

## Discussion

Our study found that both the clinical presentation of KS and clinical outcomes differ between men and women in Uganda. Women presented with lower CD4 T-cell counts at diagnosis, had frequent orofacial KS, and were less likely to have tumor-associated edema or nodular lesions than men. Women were also less likely to demonstrate clinical improvement than men.

Women in our study had significantly lower CD4 counts at KS diagnosis than men, as has been reported by others [8], [9]. The frequency of this finding across several studies suggests that greater immunosuppression may be an intrinsic part of the pathophysiology of KS in women. More severe immunosuppression due to HIV may be needed in women for KS to overcome gender-related factors, including hormonal, environmental, or genetic factors, which normally protect women against the disease. Human chorionic gonadotrophin (hCG) has been hypothesized to be a protective factor in KS development based on its inhibition of the growth KS cell lines *in vitro* [18]. However, therapeutic trials of intralesional hCG have been inconclusive and pregnancy does not affect the risk of developing KS, suggesting factors other than hCG are important [19]–[21]. Gender-based differences in immune responses, as evidenced epidemiologically by differences in rates of autoimmune disease or vaccine response in men and women [22], could also contribute to differences in KS presentation and response. Control of HHV-8, the etiologic agent of KS, may also differ in men and women, as evidenced by a cohort study of persons with HIV and KS in Uganda showing that men have higher rates of HHV-8 replication in mucosa and peripheral blood than women [23]. HHV-8 replication is in turn an essential step in the development of KS [24]–[28]. However, in US [29], [30] and Ugandan [31] cohorts, lower CD4 counts were not associated with poorer control of HHV-8 replication. Thus the interaction between immunosuppression, HHV-8 replication and development of KS in women needs to be further investigated.

We also observed that the location of KS lesions differed between men and women. Our finding that women had frequent oral KS lesions is in agreement with previous studies [9]. Notably, the presence of lesions on the hard palate was also associated with a greater hazard of improvement. It is unclear why palatal lesions would be associated with improvement, particularly since palatal KS has been identified as a marker of pulmonary KS in Uganda [32]. It is possible that persons with more severe disease offer a greater opportunity for clinicians to observe a clinical response, though we did not find an association between burden of disease as measured by number of lesions or lesion locations and response. The trend that men are more likely to present with lower extremity lesions and associated edema mirrors the typical presentation of non-HIV-associated KS, which is predominantly a disease of men. The higher likelihood of lower extremity involvement in men supports the notion that there may be gender differences in KS pathophysiology.

Male gender was significantly associated with the probability of clinical improvement. Importantly, this association remained after adjusting for KS clinical presentation, suggesting that the lower likelihood of clinical improvement in women may in part be attributed to additional factors beyond the more severe disease presentation and greater immunosuppression at KS diagnosis observed among women in our cohort. Of note, we did not identify any cases of KS immune reconstitution inflammatory syndrome (IRIS) in our cohort, and we are not aware of gender differences in IRIS risk that could account for the lower rate of KS improvement in women. A factor not evaluated in our study that could impact our findings is the type of therapy received by men and women. KS treatment usually involves administration of antiretroviral therapy and chemotherapy, but the treatment provided to men and women in our cohort may have differed due to clinical or socioeconomic factors. However, if the pathology of KS differs in men and women, it is also possible that equivalent KS treatment regimens are not equally effective in women. Future studies of KS in women should include evaluation of treatment to measure the effect of treatment on response and to determine if current therapies are equally efficacious in both genders.

Health care utilization and health-seeking behaviors may have also differed among men and women. For example, men may have been more likely to have money to pay for chemotherapy than women, perhaps contributing to the higher rate of clinical improvement we observed in men. Additionally, women tend to have greater exposure to HIV testing as routine counseling and testing has been applied to perinatal settings, and this may have allowed for both a greater number of KS cases and perhaps earlier stage disease to be identified among women. The frequency of clinic attendance after KS diagnosis could also influence the likelihood of receiving treatment or observing an “improved” response, but we found no difference in follow-up time between men and women in our cohort. Future prospective studies should also capture detailed data on health care utilization patterns to carefully evaluate their impact on KS outcomes in men and women.

Our study has several important limitations. As a retrospective chart review, our study is subject to incomplete data which may have led to misclassification of presentation or response variables, and consequently limited our ability to observe important associations. In particular, complete ACTG staging was not available for patients and there was limited assessment of visceral KS involvement in this cohort. Our classification of “improvement” relied on clinician documentation in charts, and it is possible that changes in lesion size, lesion number, or edema were not consistently documented in the medical record. Underestimating “improvement” should lead to a conservative misclassification, however, and would diminish the apparent difference in clinical improvement between men and women. Further, we could not reliably determine other important outcome measures, including mortality, because we were unable to ascertain if loss to follow up was due to death or another factor. Clinical improvement as defined by any lesion regression may not correspond to survival benefit, so it will be important to evaluate survival in future studies.

Despite these limitations, our study supports the hypothesis that gender affects the pathophysiology of HIV-associated KS. Our findings suggest that it may be possible to prevent many cases of KS in HIV-infected women if they are able to initiate ART at higher CD4 T-cell counts, as recommended by international guidelines [33], and that clinicians caring for HIV-infected women in KS-endemic areas should carefully examine the hard palate for earlier detection of KS. Prospective studies are needed to identify predictors of response and to evaluate response to treatment in women with KS, particularly in Africa where the disease burden is greatest. Such studies will not only address potential gender disparities in KS management, but will also provide an unprecedented opportunity to gain insight into the basic pathophysiology of KS by exploring gender differences in a disease that, prior to the AIDS epidemic, was almost exclusively found in men.

## References

[1] Onyango JF, Njiru A (2004). Kaposis sarcoma in a Nairobi hospital.. East Afr Med J.

[2] Parkin DM, Wabinga H, Nambooze S, Wabwire-Mangen F (1999). AIDS-related cancers in Africa: maturation of the epidemic in Uganda.. AIDS.

[3] Wabinga HR, Parkin DM, Wabwire-Mangen F, Nambooze S (2000). Trends in cancer incidence in Kyadondo County, Uganda, 1960-1997.. Br J Cancer.

[4] Chokunonga E, Levy LM, Bassett MT, Mauchaza BG, Thomas DB (2000). Cancer incidence in the African population of Harare, Zimbabwe: second results from the cancer registry 1993-1995.. Int J Cancer.

[5] Mwanda OW, Fu P, Collea R, Whalen C, Remick SC (2005). Kaposi's sarcoma in patients with and without human immunodeficiency virus infection, in a tertiary referral centre in Kenya.. Ann Trop Med Parasitol.

[6] Mbulaiteye SM, Katabira ET, Wabinga H, Parkin DM, Virgo P (2006). Spectrum of cancers among HIV-infected persons in Africa: The Uganda AIDS-Cancer Registry Match Study.. International Journal of Cancer.

[7] Wang J, Stebbing J, Bower M (2007). HIV-associated Kaposi sarcoma and gender.. Gend Med.

[8] Nasti G, Serraino D, Ridolfo A, Antinori A, Rizzardini G (1999). AIDS-associated Kaposi's sarcoma is more aggressive in women: a study of 54 patients.. J Acquir Immune Defic Syndr Hum Retrovirol.

[9] Lassoued K, Clauvel JP, Fegueux S, Matheron S, Gorin I (1991). AIDS-associated Kaposi's sarcoma in female patients.. AIDS.

[10] Benedetti P, Greco D, Figoli F, Tirelli U (1991). Epidemic Kaposi's sarcoma in female AIDS patients–a report of 23 Italian cases.. AIDS.

[11] Cooley TP, Hirschhorn LR, O'Keane JC (1996). Kaposi's sarcoma in women with AIDS.. AIDS.

[12] Mosam A, Hurkchand HP, Cassol E, Page T, Cassol S (2008). Characteristics of HIV-1-associated Kaposi's sarcoma among women and men in South Africa.. Int J STD AIDS.

[13] Meditz AL, Borok M, MaWhinney S, Gudza I, Ndemera B (2007). Gender differences in AIDS-associated Kaposi sarcoma in Harare, Zimbabwe.. J Acquir Immune Defic Syndr.

[14] WHO Obesity: preventing and managing the global epidemic..

[15] Karnofsky DABJ, M CM (1949). The clinical evaluation of chemotherapeutic agents in cancer.. Evaluation of chemotherapeutic agents.

[16] Krown S, Testa M, Huang J (1997). AIDS-related Kaposi's sarcoma: prospective validation of the AIDS Clinical Trials Group staging classification. AIDS Clinical Trials Group Oncology Committee.. J Clin Oncol.

[17] Krown SE, Metroka C, Wernz JC (1989). Kaposi's sarcoma in the acquired immune deficiency syndrome: a proposal for uniform evaluation, response, and staging criteria. AIDS Clinical Trials Group Oncology Committee.. J Clin Oncol.

[18] Lunardi-Iskandar Y, Bryant JL, Zeman RA, Lam VH, Samaniego F (1995). Tumorigenesis and metastasis of neoplastic Kaposi's sarcoma cell line in immunodeficient mice blocked by a human pregnancy hormone.. Nature.

[19] Gill PS, Lunardi-Ishkandar Y, Louie S, Tulpule A, Zheng T (1996). The effects of preparations of human chorionic gonadotropin on AIDS-related Kaposi's sarcoma.. N Engl J Med.

[20] Tavio M, Nasti G, Simonelli C, Vaccher E, De Paoli P (1998). Human chorionic gonadotropin in the treatment of HIV-related Kaposi's sarcoma.. Eur J Cancer.

[21] Rabkin CS, Chibwe G, Muyunda K, Musaba E (1995). Kaposi's sarcoma in pregnant women.. Nature 377: 21; author reply.

[22] Stanberry LR, Spruance SL, Cunningham AL, Bernstein DI, Mindel A (2002). Glycoprotein-D-adjuvant vaccine to prevent genital herpes.. N Engl J Med.

[23] Johnston C, Orem J, Okuku F, Kalinaki M, Saracino M (2009). Impact of HIV Infection and Kaposi Sarcoma on Human Herpesvirus-8 Mucosal Replication and Dissemination in Uganda.. PLoS One.

[24] Laney AS, Cannon MJ, Jaffe HW, Offermann MK, Ou CY (2007). Human herpesvirus 8 presence and viral load are associated with the progression of AIDS-associated Kaposi's sarcoma.. Aids.

[25] Laney AS, Dollard SC, Jaffe HW, Offermann MK, Spira TJ (2004). Repeated measures study of human herpesvirus 8 (HHV-8) DNA and antibodies in men seropositive for both HHV-8 and HIV.. Aids.

[26] Smith MS, Bloomer C, Horvat R, Goldstein E, Casparian JM (1997). Detection of human herpesvirus 8 DNA in Kaposi's sarcoma lesions and peripheral blood of human immunodeficiency virus-positive patients and correlation with serologic measurements.. J Infect Dis.

[27] Alagiozoglou L, Morris L, Bredell H, Martin DJ, Sitas F (2003). Human herpesvirus-8 antibodies and DNA in HIV-1 infected patients in South Africa.. Epidemiol Infect.

[28] Ziegler J, Newton R, Bourboulia D, Casabonne D, Beral V (2003). Risk factors for Kaposi's sarcoma: a case-control study of HIV- seronegative people in Uganda.. Int J Cancer.

[29] Casper C, Redman M, Huang ML, Pauk J, Lampinen TM (2004). HIV Infection and Human Herpesvirus-8 Oral Shedding Among Men Who Have Sex with Men.. J Acquir Immune Defic Syndr.

[30] Gandhi M, Koelle DM, Ameli N, Bacchetti P, Greenspan JS (2004). Prevalence of human herpesvirus-8 salivary shedding in HIV increases with CD4 count.. J Dent Res.

[31] Johnston C, Orem J, Okuku F, Kalinaki M, Saracino M (2009). Impact of HIV Infection and Kaposi Sarcoma on Human Herpesvirus-8 Mucosal Replication and Dissemination in Uganda.. PLoS ONE.

[32] Yoo D, Lee K, Munderi P, Sin K, Lee J (2005). Clinical and bronchoscopic findings in Ugandans with pulmonary Kaposi's sarcoma.. Korean Journal of Internal Medicine.

[33] WHO (2009). Rapid advice: antiretroviral therapy for HIV infection in adults and adolescents.. http://www.who.int/hiv/pub/arv/advice/en/index.html.

